# Measuring Interactions in Categorical Datasets Using Multivariate Symmetrical Uncertainty

**DOI:** 10.3390/e24010064

**Published:** 2021-12-30

**Authors:** Santiago Gómez-Guerrero, Inocencio Ortiz, Gustavo Sosa-Cabrera, Miguel García-Torres, Christian E. Schaerer

**Affiliations:** 1 Polytechnic School, National University of Asuncion, San Lorenzo 2111, Paraguay; inortiz@pol.una.py (I.O.); gdsosa@pol.una.py (G.S.-C.); cschaer@pol.una.py (C.E.S.); 2 Data Science and Big Data Lab, Universidad Pablo de Olavide, ES-41013 Seville, Spain; mgarciat@upo.es

**Keywords:** interaction, intrinsic interaction, categorical data, patterned data, multivariable correlation, gain in multiple correlation, multivariate symmetrical uncertainty

## Abstract

Interaction between variables is often found in statistical models, and it is usually expressed in the model as an additional term when the variables are numeric. However, when the variables are categorical (also known as nominal or qualitative) or mixed numerical-categorical, defining, detecting, and measuring interactions is not a simple task. In this work, based on an entropy-based correlation measure for *n* nominal variables (named as Multivariate Symmetrical Uncertainty (MSU)), we propose a formal and broader definition for the interaction of the variables. Two series of experiments are presented. In the first series, we observe that datasets where some record types or combinations of categories are absent, forming *patterns* of records, which often display interactions among their attributes. In the second series, the interaction/non-interaction behavior of a regression model (entirely built on continuous variables) gets successfully replicated under a discretized version of the dataset. It is shown that there is an interaction-wise correspondence between the continuous and the discretized versions of the dataset. Hence, we demonstrate that the proposed definition of interaction enabled by the MSU is a valuable tool for detecting and measuring interactions within linear and non-linear models.

## 1. Introduction

Correlation measures started early in the history of Statistical Science. Given two numeric variables *X* and *Y*, Pearson proposed a linear correlation based on covariance and the standard deviations of *X* and *Y* [1]. Spearman employed the same way of computation using the ranks of *X* and *Y* instead of their values and obtained a measure that is robust in the presence of outliers [2]. These initial measures dealt with a linear correlation between two variables, but a drawback is that they are limited to a pair of variables.

**Multiple Correlation**. In the multivariate world, many of the observed phenomena require a nonlinear model, and hence, a good measure of correlation should be able to detect both linear and nonlinear correlations. The so-called Coefficient of Multiple Correlation $R^{2}$ is computed in multiple regression from the square matrix $R_{xx}$ formed by all the paired correlations between variables [3]. It measures how well a given variable can be predicted using a linear function of the set of the other variables. In effect, *R* measures the linear correlation between the observed and the predicted values of the target attribute or response *Y*.

Seeking to achieve a nonlinear correlation measure for numeric variables, Viole and Nawrocki’s approach [4] builds a piecewise-linear relationship on each pair of dimensions, obtaining a non-linear correlation along with an indicator of dependence. For categorical variables, it is not so common to find a multiple correlation measure; we mention the one proposed by Colignatus [5], which is based on contingency tables and determinants.

In the Information Theory approach, several information measures have been introduced to analyze multivariate dependencies [6,7,8,9,10,11,12,13].

These multivariate information measures have been applied in fields such as physical systems [14], biological systems [15], medical data analysis [16], and neuroscience [17]. Such measures have also been applied to feature selection in order to understand how a single feature can be considered irrelevant when treated as an independent feature, but it may become a relevant feature when combined with other features through its unique and synergistic contribution [18,19].

Carrying the work forward with information theory, the symmetrical uncertainty (SU) was introduced by Arias et al. [20] based on comparison of entropies. As a natural extension, the authors of the present article have proposed the Multivariate Symmetrical Uncertainty (MSU) [18,21,22]. Both SU and MSU offer the advantage that their values range from 0 to 1, thus saving us from negative correlation values that would have no simple interpretation in the multivariate case. In addition, MSU values naturally allow the formation of groups of correlated variables, which is useful in feature selection tasks.

In feature selection, correlation has been associated with similarity and redundancy, and along with relevancy, these are the concepts most studied and analyzed [23,24,25]. However, in recent works, new concepts, such as synergy [26], interaction [16] and complementarity [27], are being studied to understand the various relationship types among features. In this context, for categorical variables, the terms correlation and interaction have been used interchangeably for some time, as in [6,7].

It is important to note that multivariate situations presenting categorical variables or a mix of categorical and numerical variables have been studied within specific areas, such as the processing of mix-type data and categorical data clustering [28,29,30]. However, these tools are applicable to observation points, whereas statistical interaction occurs between variables in any given dataset. We may see MSU or any multiple correlation measure as a tool that works in the space of random variables as opposed to the space of individual observation points.

**Interaction.** Consider a pure multivariate linear regression model of a continuous random variable *Y* explained by a set of continuous variables $X_{1},X_{2},\ldots ,X_{n}$. From here on, we adopt statistical usage whereby capital letters refer to random variables and the corresponding small case letters refer to particular values or outcomes observed. Each outcome $y_{i}$ is modeled as a linear combination of the observed variable values [31],

(1) $$y_{i}=b_{0}+b_{1}x_{1i}+b_{2}x_{2i}+\ldots +b_{n}x_{ni}$$

where $b_{i}$ is a real number. Sometimes, an additional complexity may appear, where $y_{i}$ is also dependent on the product of two or more of the variables; for example, $b_{jk}x_{ji}x_{ki}$, where 1≤j≤k≤n. In statistics, this extra term is called an *interaction* term, and it expresses how the values of $x_{ji}$ and $x_{ki}$ work together to determine $y_{i}$. An interaction term is usually the product of two or more variables, but it could also involve logs or other nonlinear functions.

The above description allows to operationalize the estimation of an interaction term in statistical regression and analysis of variance. However, a formal definition is necessary for the concept of statistical interaction that could possibly cover the case of categorical random variables as well.

Joint simultaneous participation of two or more variables that determine the value of a response can also be found in the world of categorical variables. A variable $X_{1}$ that seems irrelevant when taken in isolation with a response *Y* may be jointly relevant to that response when considered with another variable $X_{2}$; this is notably exemplified in the XOR behavior described in [22]. This is a manifestation of the interactions between categorical variables. To determine the statistical relevance of a feature with respect to a response variable, we need a suitable correlation measure for categorical variables. The detection of *n*-way interactions will become easier if the measure can also assess multivariate correlations within groups of 3, 4, or more variables, as will be shown in the following sections.

The main objective of this work is to achieve a formal definition of interaction in the statistical sense, applicable to both continuous and categorical variable models. In our first series of experiments, we discover that datasets in the form of *patterns of records* actually produce MSU correlation values lying within a subinterval of [0, 1], depending on the particular sample obtained. Thus, in this work, we use the MSU measure of correlation because its computation scheme lends itself to finding the subinterval of correlation values by simulating frequency histograms of the pattern records on a spreadsheet. We will see that for each given pattern, these values play a role in the size of interaction.

Consider two sets of variables A and C, where A⊂C. If MSU(A) < MSU(C), the added variables coming from C strengthen the dependency within the group, and we can see this strengthening as a positive interaction between variables in A and variables in C−A. In the second series of experiments, we put to the test this “cohesion boost” view of interaction in the context of classical statistical regression.

Testing the statistical significance of a categorical variable interaction by analyzing the focal predictor’s effect on the dependent variable separately for each category is common in psychological research for moderation hypotheses [32]. Thus, interaction between explanatory variables also has a crucial role across different kinds of problems in data mining, such as attribute construction, coping with small disjuncts, induction of first-order logic rules, detection of Simpson’s paradox, and finding several types of interesting rules [33].

**Contributions**. The main contribution of this paper is that it proposes a formalization of the concept of interaction for both continuous and categorical responses. Interaction is often found in Multiple Linear Regression [31] and Analysis of Variance models [34], and it is described as a departure from the linearity of effect in each variable. However, for an all-categorical-variables context, there is no definition of interaction. This work proposes a definition that is facilitated by the MSU measure and shows that it is suitable for both types of variables. The detection and quantification of interactions in any group of features of a categorical dataset is the second aim of the work.

The article begins by presenting a multivariate situation, introducing the concepts of patterned datasets and interactions, both among continuous and categorical random variables, in Section 2. Synthetic databases are then used in Section 3 to study interaction in a patterned dataset, measured as a change in the MSU value when increasing the number of variables from *j* to j+1. This experimentation allows us to propose a formal definition of interaction and how to measure it for categorical patterned data at the end of this section. In Section 4, two regression problems are presented to compare: a continuous case without interaction vs. its discretized version, and similarly, a continuous case with interaction vs. its discretized version. The appropriateness of the proposed definitions is indicated by the correspondence of computed interaction results with the coefficients estimated by the regression tool. Section 5 discusses how a linear model without significant interaction impacts a small minimum instrinsic interaction value on its discretized counterpart. Conclusions and future work are presented in Section 6.

## 2. Patterned Records and the Detection of Interactions

Let D be a population of records, each being an observation of *n* categorical variables $X_{1},\ldots ,X_{n}$. Assume no missing values in the dataset. These variables have cardinalities $c\left(X_{1}\right),\ldots ,c\left(X_{n}\right)$, each representing the number of possible categories or values in the attributes. The variety of records that may be sampled from D is given by

(2) $$V=\prod_{i=1}^{n}c\left(X_{i}\right)$$

corresponding to the number of different *n*-tuples that can be formed by combining categories in the given order.

Without the loss of generality, we assume that each row of the dataset is a record full of value; that is, no column has an empty or missing value. Hence, it is always possible to impute a value where necessary, according to a procedure of our choosing.

In practice, the *V* different types of records are not always present or do not even exist at the time a sample is taken from the field. This sort of natural incompleteness in certain datasets brings us to the notion of patterns, defined as follows.

**Definition** **1.** *An n-way pattern P is any proper subset of unique n-tuples taken from D.*

**Definition** **2.** *We say that a sample S taken from D is patterned after P if every record in S can be found in P.*

The size of the sample need not be fixed, and a given record may appear one or more times in the sample. That is, a sample may contain repeated records, for instance, when two or more individuals happen to have the same attribute values for the variables being considered.

**Figure 1:**
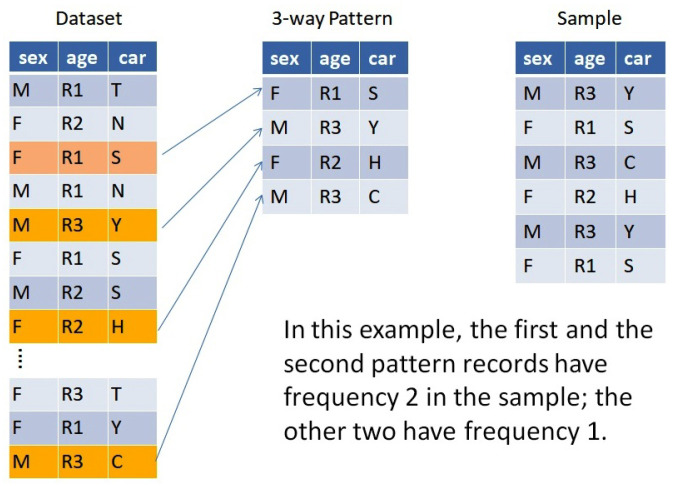
Dataset, pattern, and sample in a 3-variable example. The dataset (or population) may contain many records, of which only a sample is actually collected. *Pattern* is the name given to the set of distinct records in the sample.

**Example** **1.** *Figure 1 shows a population with 3 attributes, age, sex, and car make, which are assumed to have been recorded as a finite dataset. Four of the records exemplify a pattern. Of the many different possible samples, the 6-record sample in the figure happens to follow this pattern.*

By focusing attention on a certain pattern P, we can study the behavior of correlations across the many samples that follow P. For that purpose, we use the Multivariate Symmetrical Uncertainty (MSU) to measure correlations in samples of categorical variables. MSU is a recently developed entropy-based correlation measure formally presented in [18]. For the reader’s convenience, we recall here the definition of MSU as well as its main properties we are going to need.

**Definition of MSU.** Let $X_{i}$ be a categorical (discrete) random variable with cardinality $c(X_{i})\in N$, and possible values $x_{ij}$ with $j=\{1,\ldots ,c\left(X_{i}\right)\}$. Let $P(X_{i})$ be its probability mass function. The entropy *H* of the individual variable $X_{i}$ is a measure of the uncertainty in predicting the value of $X_{i}$ and is defined as:

(3) $$H\left(X_{i}\right):=-\sum_{j}P\left(x_{ij}\right)\log_{2}\left(P\left(x_{ij}\right)\right),$$

where $P(x_{ij})$ is the prior probability of the value $x_{ij}$ of $X_{i}$. This can be expressed in a simpler manner as

(4) $$H\left(X_{i}\right):=-\sum_{x_{i}}P\left(x_{i}\right)\log_{2}\left(P\left(x_{i}\right)\right).$$

where, as indicated in the Introduction, the small case $x_{i}$ represents the observed values of $X_{i}$. $H(X_{i})$ can also be interpreted as a measure of the *amount of information* a discrete random variable $X_{i}$ produces or the *variety* inherent to $X_{i}$ [35].

Given a set of *n* random variables $X_{1},\ldots ,X_{n}$ with a joint probability mass function $P(x_{1},\ldots ,x_{n})$, their joint entropy is defined as [21]

(5) $$H\left(X_{1},\ldots ,X_{n}\right)=H\left(X_{1:n}\right):=-\sum_{x_{1}}\ldots \sum_{x_{n}}P\left(x_{1},...,x_{n}\right)\log_{2}\left[P\left(x_{1},\ldots ,x_{n}\right)\right]$$

The Multivariate Symmetrical Uncertainty is then defined as follows:

(6) $$MSU\left(X_{1:n}\right):=\frac{n}{n-1}\left[1-\frac{H(X_{1:n})}{\sum_{i=1}^{n}H\left(X_{i}\right)}\right].$$

That is, the joint entropy (5) is compared with the sum of individual entropies (4) by way of a ratio. This measure of correlation and its properties were presented in [21]. Some key properties are:

(a) The MSU values are in the unit range, $MSU\left(X_{1:n}\right)\in \left[0,1\right]$;
(b) Higher values in the measure correspond to higher correlation among variables, i.e., a value of 0 implies that all variables are independent while a value of 1 corresponds to a perfect correlation among variables; and
(c) MSU detects linear and non-linear correlations between any mix of categorical and/or discretized numerical variables.

We perform most of our MSU calculations on a spreadsheet for easier handling and better understanding of the pattern’s behavior.

**Interaction among continuous variables.** Let us begin with a two-variable example. Consider the regression model

(7) $$y=b_{0}+b_{1}x_{1}+b_{2}x_{2}+b_{12}x_{1}x_{2}$$

where $b_{0}$, $b_{1}$, $b_{2}$, and $b_{12}$ are parameters to be estimated using the sample data. If $b_{12}=0$, we have a linear model, with additive effects from $x_{1}$ and $x_{2}$. If $b_{12}$ differs from 0 (with significance testable via *p*-values in the regression summary output), we say that there is *interaction* among the three variables. With a nonzero interaction term, the individual contributions of $x_{1}$ and $x_{2}$ are still present, but obtaining the predicted *y* value also depends on a nonlinear function of both of them—in this case, their product $x_{1}x_{2}$.

Naturally, models with interaction may have more than two independent variables and possibly more than one interaction term. Each interaction term may have other types of nonlinear functions, containing, for instance, powers or logs of the independent variables.

To sum up, regression models, such as Equation (7), and analysis of variance models with continuous responses, include a coefficient indicating the strength of association between each variable or combination and the response. This allows detecting interaction if it is postulated as part of the model.

**Interaction among categorical variables.** Categorical or nominal features are also employed to build various types of multivariate models with a categorical response. Established modeling techniques include, for example, Categorical Principal Components Analysis, Multiple Correspondence Analysis, and Multiple Factor Analysis [36]. In this realm, we can measure the strength of association between two, three, or more categorical variables by means of both MSU and the study of patterns’ behavior; this will, in turn, allow us to detect interactions.

## 3. Simulations Using Patterns

Given a pattern P of records, the simplest sample patterned after P is the one having each category combination appearing just once (single-frequency sample). However, it is also possible to obtain samples with different frequencies on each category. Since MSU estimations from samples are based on the actual frequencies found, each of these different samples will have a specific MSU estimate.

This section reports simulation experiments performed on records patterned after well-known logic gates (also known as truth tables). There is no reason for choosing logic gates other than their simplicity, which may help uncover specific characteristics of the interaction behavior. Simulations seek to gain insight on the sensitivity of our MSU multiple correlation estimate under a variety of sampling scenarios. Later in the paper we will present patterns induced by “real-life” data collected as continuous variables.

### 3.1. Three-Way XOR

The three-way Exclusive OR pattern contains four distinct records. Assuming that the four record types are equally likely (probability 0.25 on each record), its resulting MSU is just 0.5.

However, samples with more than four records also allow unequal likelihoods, and we observe that the computed sample MSU increases. Intuitively, this happens because some combinations of *A* and *B* co-occur with their respective *C* values more frequently than other combinations, inducing more correlation. For example, the probability vector $\left(0.25;1\times 10^{-80};1\times 10^{-80};0.75\right)$ gives an MSU of 0.75. Table 1 shows both calculation scenarios.

Every simulation run amounts to computing the value of function MSU based on *k* probability or frequency values, where *k* is the number of rows in the pattern under consideration. In the three-way XOR, we have *k* = 4. By varying some or all of the *k* values in the column of frequencies P(X), the MSU value is modified; we want to find the *k* probabilities P(X) that produce the minimum and the maximum MSU values.

### 3.2. Four-Way XOR

The four-way Exclusive OR pattern contains eight distinct records. If the eight of them are equally likely, the MSU for the plain pattern (three-variables plus the XOR column) is exactly 1/3.

Again, samples of more than eight cases allow unequal likelihoods, increasing the MSU of the sample. With seven very small P(X) values and one large P(X), we observe a maximum four-way MSU value of almost 0.75.

Table 2 shows both calculation scenarios.

### 3.3. Four-Way AND

In the four-way AND pattern, the three variables *A*, *B* and *C* must be True (one of eight cases) in order for AND to be true. The other seven cases give a False on the AND column; so, nearly regardless of the combination of values, AND is false. That is, the correlation is weak.

With eight equally likely records, the MSU for the plain pattern (three-variable plus the AND function) is 0.2045.

With unequal likelihoods, the sample MSU increases again. The maximum MSU is 1 when P(X) is (0.2; $1\times 10^{-80}$; …; $1\times 10^{-80}$; 0.8) or any permutation thereof.

See Table 3 displaying the computation for equally likely records.

From these examples, one might think that equiprobable sampling scenarios always produce a minimum MSU value. However, this is not always true as two of the OR cases in Table 4 and an example later on will demonstrate.

### 3.4. Further Simulations

Table 4 shows a number of similar experiments performed, using a variety of patterns and variable cardinalities. Here is a comparison of the MSU behavior in the previous and other specific patterns.

### 3.5. Discussion and Interpretation of Results

In multiple regression and analysis of variance with a numeric response, each term’s coefficient gives an indication of the strength of association in the positive or negative direction. For instance, in Equation (7), we say that there is interaction if the coefficient of the (nonlinear) product term is different from 0.

When the response is categorical, the MSU correlation measure for each variable or combination of variables indicates how strong an association is; hence, we can use MSU to establish a parallel with the numeric responses. For example, in Table 4, the second OR row has bivariate correlations of 0.344 for AC and BC, whereas the correlation for the ABC combination is 0.433. It is reasonable for taking MSU as a basis for defining interactions between categorical variables.

**Definition** **3.** *Let A, B, and C be any three categorical variables in a dataset. The **gain in multiple correlation*** *obtained by adding B (or BC) to AC, forming ABC is defined as*

G(AC,ABC):=MSU(ABC)−MSU(AC).

Referring to the above Table 4 and taking the second OR row as an example,

(8) G(AC,ABC)=MSU(ABC)−MSU(AC)=0.433−0.344=0.089

is the gain in multiple correlation. Note that *G* also equals MSU(ABC)−MSU(BC). Let us now define the interaction that can be found when one increases dimensionality (the number of variables) of the dataset from *j* to *k*.

**Definition** **4.** *Consider a dataset D of n categorical random variables. Let A = $\left\{A_{1},\ldots ,A_{j}\right\}$ and C = $\left\{C_{1},\ldots ,C_{k}\right\}$ be sets of variables in D, with 2≤j<k≤n and A⊂C. We define the **interaction*** *among variables in C on top of j variables as*

*min${}_{j}$ G(A, C) = min${}_{j}$ [MSU(C) − MSU(A)] = MSU(C) − max${}_{j}$ MSU(A).*

Thus, the interaction on top of *j* variables is the smallest gain in the multiple correlation found by adding to A the k−j variables of the complement C−A over all possible *j*-element sets A⊂C.

It can be seen that the reason to choose the smallest gain in multiple correlation is that this lowest gain is achieved by finding the *j*-variable subset A that has maximum group correlation.

Note that *M* = max${}_{j}$ MSU(A) is the largest known correlation of *j* variables included in C. By adding k−j more variables, the resulting global correlation may be larger or smaller than *M*. If larger, the interaction G(A, C) is positive; if smaller, the interaction is negative.

**Example** **2.** *Example: XOR revisited. Let $X_{1}$, $X_{2}$, and $X_{3}$ be three variables in a XOR pattern of equally likely records. For this pattern, j = 2, k = 3, $A=\{X_{1},X_{2}\}$, and $C=\{X_{1},X_{2},X_{3}\}$. The interaction among the three variables in C from adding variable $X_{3}$ to A is*

(9) $$min_{j}\;G\left(A,C\right)=MSU\left(C\right)-max_{2}\;MSU\left(A\right)=0.5-0=0.5.$$

In positive interaction, group correlation is strengthened by the added variables; in negative interaction, group correlation is weakened. When modeling, we want to identify groups of variables or factors that work in the same direction; hence, variables that bring in a negative interaction would not usually be included in a group by a researcher.

**Complexity of Interaction Calculation**. The following approach is module-based. In a dataset of *r* observation rows on *n* variables, let $c_{i}$ be the cardinality of the *i*-th variable. The two sets being considered are C with *k* variables and A with *j* variables, such that A⊂C.

The cost of obtaining MSU(C), where C is a *k*-variable subset of the *n* variables in the dataset, has components of three types:

- Entropy of each attribute—For each attribute $X_{i}$, there are $c_{i}$ frequencies $P(x_{i})$ and $c_{i}$ logarithms $\log_{2}\left(P\left(x_{i}\right)\right)$, which are multiplied according to Equation (4), giving $3c_{i}$ operations. This is conducted *k* times, giving $3\sum_{1}^{k}c_{i}$.
- Joint entropy of all *k* attributes—There are $\prod_{1}^{k}c_{i}$ combinations of values, and for each one of them, the frequencies as well as their logarithms are calculated and multiplied according to Equation (5), giving $3\prod_{1}^{k}c_{i}$ operations. This is conducted one time.
- msucost(C)—Using Equation (6), the costs of the numerator and the denominator are added, followed by one division and one difference. This gives $3\sum_{1}^{k}c_{i}+3\prod_{1}^{k}c_{i}+2$ operations.

For the cost of obtaining each of the MSU(A), we only need to consider that we have *j* attributes instead of *k*. In order to obtain the maximum value of Definition 4, we assume that the MSU values for all subsets A need evaluation. Therefore, the cost *b* of running the algorithm is

(10) $$\begin{array}{cc} b & =msucost\left(C\right)+\left(\begin{array}{c} k\\ j \end{array}\right)\cdot msucost\left(A\right)\\ \; & =3\sum_{1}^{k}c_{i}+3\prod_{1}^{k}c_{i}+2+\left(\begin{array}{c} k\\ j \end{array}\right)\cdot \left(3\sum_{1}^{j}c_{i}+3\prod_{1}^{j}c_{i}+2\right) \end{array}$$

Since individual entropies are used over and over, each of them needs only be calculated once and then saved to a disk or temporary memory during the calculation. Thus, the term $3\sum_{1}^{j}c_{i}$ can be dropped, and we have

(11) $$\begin{array}{cc} b & =3\sum_{1}^{k}c_{i}+3\prod_{1}^{k}c_{i}+2+\left(\begin{array}{c} k\\ j \end{array}\right)\cdot \left(3\prod_{1}^{j}c_{i}+2\right) \end{array}$$

Thus, *b* depends on $c_{i}$, the number of categories of each variable, and the relative sizes of *k* and *j*. Often in statistics, *k* and *j* differ by only 1 as the researcher wants to know how much interaction is due to adding one variable. The number of rows *r* in the dataset is hidden within the $c_{i}$ since each $P(x_{i})$ is computed as a category count divided by *r*. Further economies in the calculation effort may be achieved by organizing the joint entropies of the A sets in a hierarchical fashion.

We know that the calculated values of MSU and of any interaction measure depend on the specific sample obtained. Hence, when several samples are taken from the same patterned dataset, MSU values may vary within the interval [0, 1]. Actually, the minimum and maximum MSU values for each pattern as found through simulations (Table 4) indicate that the sample MSU often ranges over a sub-interval of [0, 1]. A primary interest is the minimum value that the MSU can attain, so we formally address this situation in the following theorem, which is based on the numerator being smaller than the denominator in the MSU formula (6).

**Theorem** **1.** *Consider a categorical patterned dataset such that the joint entropy of all n variables is strictly less than the sum of their n individual entropies, and let M be the set of values attained by the MSU measure. Then, the minimum value of M over all possible frequencies observable in the pattern is a positive value $M_{L}>0$.*

**Proof.**  We refer to the proof of Lemma 4.3 in [18]. From the final line of that proof,

(12) $$MSU\left(X_{1:n}\right)\geq E\left(\hat{R}\right)>0,$$

where $\hat{R}$ is the natural estimate of MSU obtained by the quotient between the estimate of the numerator and the estimate of the denominator.

The Lemma also implies from its proof that the last inequality is strict as long as $H\left(X_{1:n}\right)<\sum_{i=1}^{n}H\left(X_{i}\right)$, which is the initial condition in this Theorem. Therefore, $M_{L}>0$. □

The minimum value $M_{L}$ being strictly positive for a categorical pattern allows the possibility of finding some interactions of a positive sign. Note that a non-patterned dataset (where all category combinations are present) may also have a positive $M_{L}$. However, as patterned sets that satisfy the Theorem 1 condition are so common in the real world, it is important to provide evidence that it is plausible to look for interactions in patterned datasets where $M_{L}>0$.

Our simulation procedure in the previous four sections consisted of keeping a pattern fixed and then running different sampling scenarios under that pattern. Through this somewhat extreme choice of patterns, it is observed that every *n*-variable pattern is characterized by a lower MSU bound $M_{L}$ and an upper MSU bound $M_{U}$.

In practice, most of the time we only get to see one sample for each dataset, and from this sample, we obtain a point estimation of *G*, the gain in multiple correlation. In general, if further samples from the given pattern were available, *G* would have varied from one sample to another. Although *M* in the above theorem can be seen as a continuous function of *k* variables, where *k* is the number of rows in the pattern, an algebraic or calculus procedure to find its global minimum and maximum may be cumbersome. However, with some computing power, we can find $M_{L}$ and $M_{U}$ via simulation runs.

Definition 4 provides a simple way to compute the interaction due to increasing the number of dimensions considered in a given sample. However, the interaction calculated at $M_{L}$ may or may not also be the minimum of the interaction values. This distinction can be expressed in the following

**Definition** **5.** *Consider a pattern P of n categorical variables, and let $M_{L}$ be the minimal value of the MSU measure when considering all n variables. If the interaction calculated at $M_{L}$ is also the minimum $I_{L}$ of all interaction values, we say that $I_{L}=M_{L}$ is the* ***intrinsic interaction*** *due to pattern P.*

The difference $M_{U}-M_{L}$ can be considered an additional correlation induced by the variation in relative frequencies from configuration $M_{L}$ to configuration $M_{U}$.

## 4. Comparison with Interaction on Continuous Variables

We now want to apply our method to a model from real life comprised of all-continuous variables. To do so, we consider the data in Table 5, which was taken from [37], and shows among various body measurements, the skinfold thickness (*st*) and the midarm circumference (*mc*) proposed as possible predictors of body fat (*bf*). It is also desired to find whether there is any evidence of interactions among the three variables. Skinfold thickness and midarm circumference have been centralized with respect to their means.

Let us start with a two-variable regression model of the form

(13) bf=k+a·st+b·mc+c·st·mc

where *k*, *a*, *b*, and *c* are parameters to be estimated.

The regression model that fits the data is:

(14) bf=20.375+0.9815·st−0.4234·mc+0.0226·st·mc,

with the coefficient of multiple determination $r^{2}$ = 0.7945 indicating that the data are quite close to the fitted regression line. These results were obtained using an online regression calculator [38]. The summary table from the calculator (not shown here) informs that the interaction term st·mc is not significant in this case, with a *p*-value of 0.4321, which makes the term negligible.

Regression variables are usually continuous, but their values may be the expression of underlying patterns. In order to detect patterns in the dataset, we can discretize the variables to enable the calculation of the MSU. We expect that the implied $M_{L}$ value will correspond to the interaction found by the model.

The adopted strategy is as follows.

- Discretize *bf*, *st* and *mc*;
- Take as pattern the set of distinct observed records, discretized;
- Simulate sampling scenarios to find $M_{L}$;
- Check whether the $M_{L}$ value reveals interactions.

### 4.1. Discretization

The discretization of *bf*, st, and mc into three categories according to their numeric value (low/medium/high) each, using percentiles (0, 33, 67, 100) as the cutoff points, gives us an all-categorical-variable database, as shown on Table 6. Under this discretization, the correlation from the sample is MSU(dst, dmc, dbf) = 0.3667.

Some duplicates can be seen among these 20 records. By removing duplicates, we will have a pattern that can be analyzed.

### 4.2. Seeking Interaction in the Pattern

Pattern 1—the 13 unique records obtained from the above 20 records implied by this database—is shown below (Table 7). A simulation of sampling scenarios leads to $M_{L}$ = 0.236828 as the lowest value of MSU. This is even lower than in the equiprobable configuration, whose MSU is 0.32646521.

Thus, the original data presented, with a regression model of no significant interaction term of the multiplicative type st·mc, maps to a discretized dataset whose $M_{L}$ value is 0.23683.

### 4.3. Creating Ad Hoc Interaction

**Figure 2:**
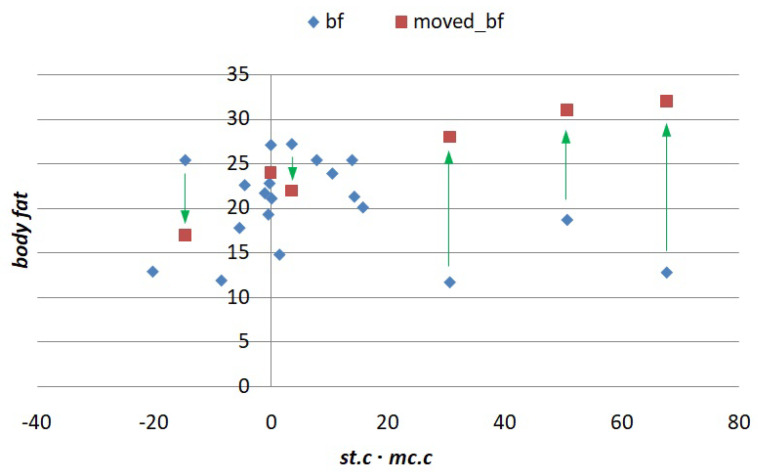
Moving a few body fat data points to produce an interaction: On a graph of *bf* as a function of product st.c·mc.c, six points were moved to induce interaction in the linear regression.

In order to exhibit the st·mc interaction, we modify some values on the *bf* column so that they follow their corresponding product term, seeking to display a more definite trend. This is accomplished by plotting *bf* against st·mc and dragging some points up or down to make the graph more linear and less horizontal. For convenience in constructing the graph, we use transformed versions of st.c and mc.c centralized with respect to their means. The new, modified points are shown in Figure 2, with arrows pointing at the squares that will replace the original diamonds.

The data table with modified points (3, 7, 12, 13, 15, and 18) is shown in Table 8.

Thus, the model becomes

(15) bf=19.9453+0.4108·st−0.2549·mc+0.2265·st·mc

with $r^{2}$ = 0.8055. This time the interaction term is significant as per the summary table, with a *p*-value very close to 0.

### 4.4. Discretizing the Modified Data

Again, we discretize *bf*, st, and mc into three categories each. Six *bf* values were manually modified, most of them being increased, so that percentiles (0, 33, 67, 100) recomputed on *bf* produce slightly higher interquartile limits or cutoff values for this discretization. The resulting categorical database is shown in Table 9. A starred *dbf* value indicates that its underlying numerical value had been modified to yield interaction detected in the model of Equation (15). Other *dbf* values are marked with an ^o^ exponent, meaning that they have been recategorized just because of modified cutoff values. All this can be verified by comparing Table 9 with Table 6.

### 4.5. Interaction in the New Pattern

Once again, the removal of duplicate records produces a pattern for analysis. The implied Pattern 2 shown on Table 10 through simulation of sampling scenarios leads us to find $M_{L}$ = 0.300573. This higher $M_{L}$ value also means that Pattern 2 can accommodate a larger interaction than Pattern 1. This is indeed the case, as shown by Table 11.

We have defined interaction as the difference between the MSU computed on a “large” set of variables and the MSU of one of its proper subsets (Definition 4). This comparison between patterns exemplifies MSU’s ability to detect levels of interaction. Pattern 2 displays higher interaction values at the three cases being simulated. As for $M_{L}$, the low $M_{L}$ value of 0.237 in Pattern 1 could be interpreted as a possibly weak form of interaction, perhaps of a non-multiplicative type. That is, interaction could be based on an expression different from st·mc, and in that case, it will not be correctly captured by this particular regression model in use.

## 5. Discussion on $M_{L}$ and Linear Models

The body fat example shows that a linear model with no significant interaction tends to have a small $M_{L}$ value compared to a model whose data has revealed interaction.

In a three-way XOR pattern with equal frequencies, it is easy to check that any two of the variables have no correlation with the third one, giving MSU(*A*, *C*) = MSU(*B*, *C*) = 0. That is, *A* and *B* are *independent* from *C*. However, when we consider the full three-way pattern the MSU(*A*, *B*, *C*) = $M_{L}$ = 0.5. Thus, it is fair to say that 0.5 is the *intrinsic interaction* due to the XOR pattern.

In the body fat example with the frequencies as first found in Pattern 1, if we look at variables pairwise, we have MSU(dst, dbf) > 0 and MSU(dmc, dbf) > 0 (as shown in Table 11). That is, both dst and dmc are relevant to dbf as opposed to the XOR example. When we simulate the behavior of the three-way Pattern 1, the $M_{L}$ value found is 0.236828. In this case, we can only say that 0.236828 represents the minimal three-way correlation due to Pattern 1, where variables dst and dmc are not independent but *relevant* to dbf.

As for Pattern 2, its $M_{L}$ value of 0.300573 indicates that, with the same values for independent variables and some modified values in the response, interaction is more visible. Furthermore, this follows the trend of a larger interaction coefficient in the regression model of Equation (15).

We see that there exists a connection between the size of $M_{L}$ and the size of interaction. Let P1 and P2 be patterns on the same variable set *X*, obtained by discretization of data. If P1 corresponds to the data of a regression model R1 without an interaction term, and P2 corresponds to the data of a regression model R2 with the addition of at least one significant interaction term, then the $M_{L}$ value computed for P1 is smaller than the $M_{L}$ value computed for P2.

Additional experimentation and comparisons are needed to provide more solid ground to the stated connection. For example, statistical regression models with more complex interaction terms and statistical models other than regression should be tested for comparability of interaction behavior with their corresponding categorical patterns.

## 6. Conclusions and Future Work

The concept of interaction for datasets of *n* categorical or discretizable variables was formalized (Definitions 3 and 4). The presented method detects *n*-way categorical interactions by finding the smallest gain in multiple correlations between the set of *n* variables and all of its proper subsets containing *j* variables each, where n>j. Since the method is applicable to both patterned and non-patterned datasets, the second goal mentioned in the Introduction is also fulfilled.

In model construction or during feature selection tasks, the discovered interactions can help improve heuristics, guide explorations, and attain better results. The discovery of interactions may depend on the adopted discretization scheme for continuous variables or on whether discretization is simple or supervised by response values. This deserves more study.

From the point of view of observational statistics and linear models with a numeric response, interaction is a way for nature to not follow a linear behavior all the time. Interaction is actually a frequent phenomenon, backed by the fact that the strict inequality premise for Theorem 1 is not rare in practice. Many times we can observe interaction as an extra term in an extended linear model, but often, its size is not large compared to the direct effect of relevant variables, and it is disregarded for model simplicity. Hence, suitable criteria are needed to decide on the statistical significance of an interaction, once it has been detected.

## Figures and Tables

**Table 1:** MSU values of 3-way XOR: minimum of 0.5 and maximum of 0.75. Here C=A⨁B where ⨁ represents the XOR operation.

3-Way Collective					3-Way ABC	1-Way A	1-Way B	1-Way C
*A*	*B*	*C*	*X*	P(X)	P(X) logP(X)	P(X) logP(X)	P(X) logP(X)	P(X) logP(X)
0	0	0	000	0.25	−0.5			
0	1	1	011	0.25	−0.5	−0.5	−0.5	−0.5
1	0	1	101	0.25	−0.5			
1	1	0	110	0.25	−0.5	−0.5	−0.5	−0.5
H(X)					2	1	1	1
MSU					0.5			
**3-Way Collective**					**3-Way ABC**	**1-Way A**	**1-Way B**	**1-Way C**
*A*	*B*	*C*	*X*	P(X)	P(X) logP(X)	P(X) logP(X)	P(X) logP(X)	P(X) logP(X)
0	0	0	000	0.25	−0.5			
0	1	1	011	1.00 × 10${}^{-80}$	−2.66 × 10${}^{-78}$	−0.5	−0.31	−5.30 × 10${}^{-78}$
1	0	1	101	1.00 × 10${}^{-80}$	−2.66 × 10${}^{-78}$			
1	1	0	110	0.75	−0.311	−0.311	−0.5	0.
H(X)					0.811	0.811	0.811	5.30 × 10${}^{-78}$
MSU					0.75			

**Table 2:** MSU values of the 4-way XOR with a minimum of 1/3 and a maximum of 0.746. Here D=A⨁B⨁C.

4-Way Collective						4-Way ABCD	1-Way A	1-Way B	1-Way C	1-Way D
*A*	*B*	*C*	*D*	*X*	P(X)	P(X) logP(X)	P(X) logP(X)	P(X) logP(X)	P(X) logP(X)	P(X) logP(X)
0	0	0	0	0000	0.125	−0.375				
0	0	1	1	0011	0.125	−0.375				
0	1	0	1	0101	0.125	−0.375				
0	1	1	0	0110	0.125	−0.375	−0.5	−0.5	−0.5	−0.5
1	0	0	1	1001	0.125	−0.375				
1	0	1	0	1010	0.125	−0.375				
1	1	0	0	1100	0.125	−0.375				
1	1	1	1	1111	0.125	−0.375	−0.5	−0.5	−0.5	−0.5
H(X)						3	1	1	1	1
MSU						0.333				
*A*	*B*	*C*	*D*	*X*	P(X)	P(X) logP(X)	P(X) logP(X)	P(X) logP(X)	P(X) logP(X)	P(X) logP(X)
0	0	0	0	0000	1.000	0.000				
0	0	1	1	0011	1.00 × 10${}^{-80}$	−2.66 × 10${}^{-78}$				
0	1	0	1	0101	1.00 × 10${}^{-80}$	−2.66 × 10${}^{-78}$				
0	1	1	0	0110	1.00 × 10${}^{-80}$	−2.66 × 10${}^{-78}$	0.0	0.0	0.0	0.0
1	0	0	1	1001	1.00 × 10${}^{-80}$	−2.66 × 10${}^{-78}$				
1	0	1	0	1010	1.00 × 10${}^{-80}$	−2.66 × 10${}^{-78}$				
1	1	0	0	1100	1.00 × 10${}^{-80}$	−2.66 × 10${}^{-78}$				
1	1	1	1	1111	1.00 × 10${}^{-80}$	−2.66 × 10${}^{-78}$	−1.06 × 10${}^{-77}$	−1.06 × 10${}^{-77}$	−1.06 × 10${}^{-77}$	−1.06 × 10${}^{-77}$
H(X)						−1.86 × 10${}^{-77}$	−1.06 × 10${}^{-77}$	−1.06 × 10${}^{-77}$	−1.06 × 10${}^{-77}$	−1.06 × 10${}^{-77}$
MSU						0.746				

**Table 3:** MSU values of the 4-way AND show a minimum of 0.2045 and a maximum of 1. Here, D=A∧B∧C.

4-Way Collective						4-Way ABCD	1-Way A	1-Way B	1-Way C	1-Way D
*A*	*B*	*C*	*D*	*X*	P(X)	P(X) logP(X)	P(X) logP(X)	P(X) logP(X)	P(X) logP(X)	P(X) logP(X)
0	0	0	0	0000	0.125	−0.375				
0	0	1	1	0011	0.125	−0.375				
0	1	0	1	0101	0.125	−0.375				
0	1	1	0	0110	0.125	−0.375	−0.5	−0.5	−0.5	−0.169
1	0	0	1	1001	0.125	−0.375				
1	0	1	0	1010	0.125	−0.375				
1	1	0	0	1100	0.125	−0.375				
1	1	1	1	1111	0.125	−0.375	−0.5	−0.5	−0.5	−0.375
H(X)						3	1	1	1	0.544
MSU						0.205				

**Table 4:** Comparative behavior of MSU for some patterns.

Name	*n*	*c*	*k*	Probab Distribution	Partial MSU Values	Global MSU
XOR	3	2	4	Equal likelohoods	MSU(AC) = 0	MSU(ABC) = 0.5
					MSU(BC) = 0	
	3	2	4	0.25; 1.00 ×$10^{-80}$; 1.00 ×$10^{-80}$; 0.75	MSU(AC) = 0	MSU(ABC) = 0.75
					MSU(BC) = 0	
XOR	4	2	8	Equal likelihoods	MSU(AD) = 0	MSU(ABCD) = 0.333
					MSU(BD) = 0	
					MSU(CD) = 0	
	4	2	8	1; 1.00 ×$10^{-80}$; 1.00 ×$10^{-80}$; …	MSU(AD) = 0.371	MSU(ABCD) = 0.746
					MSU(BD) = 0.371	
					MSU(CD) = 0.371	
AND	3	2	4	Equal likelihoods	MSU(AC) = 0.258	MSU(ABC) = 0.433
					MSU(CD) = 0.258	
	3	2	4	0.25; 1.00 ×$10^{-21}$; 1.00 ×$10^{-21}$; 0.75	MSU(AC) = 0.75	MSU(ABC) = 1
					MSU(CD) = 0.75	
AND	4	2	8	Equal likelihoods	MSU(AD) = 0.179	MSU(ABCD) = 0.205
					MSU(BD) = 0.179	
					MSU(CD) = 0.179	
	4	2	8	0.2; 1.00 ×$10^{-80}$; …; 1.00 ×$10^{-80}$; 0.8	MSU(AD) = 1	MSU(ABCD) = 1
					MSU(BD) = 1	
					MSU(CD) = 1	
OR	3	2	4	1.00 ×$10^{-21}$; 0.1; 1.00 ×$10^{-21}$; 0.9	MSU(AC) = 0	MSU(ABC) = 0
					MSU(BC) = 0.654	
	3	2	4	Equal likelihoods	MSU(AC) = 0.344	MSU(ABC) = 0.433
					MSU(BC) = 0.344	
	3	2	4	0.4; 1.00 ×$10^{-21}$; 1.00 ×$10^{-21}$; 0.6	MSU(AC) = 1	MSU(ABC) = 1
					MSU(BC) = 1	
OR	4	2	8	1.00 ×$10^{-80}$; 0.001; 0.001;	MSU(AD) = 0	MSU(ABCD) = 0.005
				0.009; 0.01; 0.125;	MSU(BD) = 0	
				0.125; 0.729	MSU(CD) = 0	
	4	2	8	Equal likelihoods	MSU(AD) = 0.179	MSU(ABCD) = 0.205
					MSU(BD) = 0.179	
					MSU(CD) = 0.179	
	4	2	8	0.2; 1.00 ×$10^{-80}$; …; 1.00 ×$10^{-80}$; 0.8	MSU(AD) = 1	MSU(ABCD) = 1
					MSU(BD) = 1	
					MSU(CD) = 1	
A∧notB	3	2	4	1.00 ×$10^{-21}$; 0.25; 1.00 ×$10^{-21}$; 0.75	MSU(AC) = 0	MSU(ABC) = 0
					MSU(BC) = 0.654	
	3	2	4	1.00 ×$10^{-21}$; 1.00 ×$10^{-21}$; 0.1; 0.9	MSU(AC) = 0	MSU(ABC) = 0.75
					MSU(BC) = 1	

*n* = Number of attributes; *c* = Cardinality of each attribute (all of them equal *c*); *k* = Number of record configurations in sample.

**Table 5:** Original Body Fat Data.

*#*	st.c	mc.c	bf
1	−5.805	1.48	11.9
2	−0.605	0.58	22.8
3	5.395	9.38	18.7
4	4.495	3.48	20.1
5	−6.205	3.28	12.9
6	0.295	−3.92	21.7
7	6.095	−0.02	27.1
8	2.595	2.98	25.4
9	−3.205	−4.42	21.3
10	0.195	−2.82	19.3
11	5.795	2.38	25.4
12	5.095	0.68	27.2
13	−6.605	−4.62	11.7
14	−5.605	0.98	17.8
15	−10.705	−6.32	12.8
16	4.195	2.48	23.9
17	2.395	−1.92	22.6
18	4.895	−3.02	25.4
19	−2.605	−0.52	14.8
20	−0.105	−0.12	21.1

**Table 6:** Original Body Fat Data discretized.

*#*	dst	dmc	dbf
1	low	high	low
2	med	med	high
3	high	high	low
4	high	high	med
5	low	high	low
6	med	low	med
7	high	med	high
8	med	high	high
9	low	low	med
10	med	low	med
11	high	high	high
12	high	med	high
13	low	low	low
14	low	med	low
15	low	low	low
16	high	high	high
17	med	low	med
18	high	low	high
19	low	med	low
20	med	med	med

**Table 7:** Pattern 1 from body fat regression and empirical finding of its lowest MSU value.

Pattern 1			P(X)	P(X)log(P(X))	1-Way dst	1-Way dmc	1-Way dbf
low	low	low	0.027	−0.141	−0.302	−0.360	−0.390
low	low	med	0.027	−0.141			
low	med	low	0.008	−0.054			
low	high	low	0.023	−0.126			
med	low	med	0.015	−0.093	−0.228	−0.194	−0.530
med	med	med	0.008	−0.054			
med	high	high	0.023	−0.126			
high	low	high	0.046	−0.205	−0.186	−0.209	−0.507
high	med	high	0.019	−0.110			
high	high	low	0.077	−0.285			
high	high	med	0.332	−0.528			
high	high	high	0.386	−0.530			
			Entropy:	2.448	0.716	0.763	1.428
			MSU:	0.237			

**Table 8:** Modified Body Fat Data with Interaction.

*#*	st.c	mc.c	bf.mod
1	−5.805	1.48	11.9
2	−0.605	0.58	22.8
3	5.395	9.38	31
4	4.495	3.48	20.1
5	−6.205	3.28	12.9
6	0.295	−3.92	21.7
7	6.095	−0.02	24
8	2.595	2.98	25.4
9	−3.205	−4.42	21.3
10	0.195	−2.82	19.3
11	5.795	2.38	25.4
12	5.095	0.68	22
13	−6.605	−4.62	28
14	−5.605	0.98	17.8
15	−10.705	−6.32	32
16	4.195	2.48	23.9
17	2.395	−1.92	22.6
18	4.895	−3.02	17
19	−2.605	−0.52	14.8
20	−0.105	−0.12	21.1

**Table 9:** Modified Body Fat Data discretized. Superscript symbol *o* denotes recategorized data because of modified cutoff values. Superscript symbol * denotes underlying numerical value modified to produce interaction.

*#*	dst	dmc	dbf
1	low	high	low
2	med	med	med ^o^
3	high	high	high *
4	high	high	low ^o^
5	low	high	low
6	med	low	med
7	high	med	high *
8	med	high	high
9	low	low	med
10	med	low	low ^o^
11	high	high	high
12	high	med	med *
13	low	low	high *
14	low	med	low
15	low	low	high *
16	high	high	high
17	med	low	med
18	high	low	low *
19	low	med	low
20	med	med	med

**Table 10:** Pattern 2 from body fat regression and empirical finding of its lowest MSU value.

Pattern 2			P(X)	P(X)log(P(X))	1-Way dst	1-Way dmc	1-Way dbf
low	low	med	0.04	−0.185	−0.523	−0.521	−0.468
low	low	high	0.06	−0.244			
low	med	low	0.08	−0.292			
low	high	low	0.13	−0.383			
med	low	low	0.06	−0.244	−0.435	−0.494	−0.423
med	low	med	0.03	−0.152			
med	med	med	0.03	−0.152			
med	high	high	0.05	−0.216			
high	low	low	0.11	−0.350	−0.491	−0.515	−0.514
high	med	med	0.06	−0.244			
high	med	high	0.07	−0.269			
high	high	low	0.18	−0.445			
high	high	high	0.1	−0.332			
			Entropy:	3.506	1.449	1.530	1.406
			MSU:	0.301			

**Table 11:** Comparative behavior of MSU and interaction for two discretized patterns.

Name	*n*	*c*	*k*	Record Frequencies	Partial MSU Values	Global MSU	Interaction
Pattern1	3	3	13	7, 7, 2, 6, 4, 2	MSU(dst, dbf) = 0.142	MSU(dst, dmc, dbf) = 0.237	0.095
				2, 6, 12, 5, 20, 86, 100	MSU(dmc, dbf) = 0.012		
	3	3	13	2, 1, 2, 2, 3, 1, 1, 1, 1, 2, 1, 1, 2	MSU(dst, dbf) = 0.441	MSU(dst, dmc, dbf) = 0.367	−0.074
				(original observations)	MSU(dmc, dbf) = 0.097		
	3	3	13	Equal frequencies	MSU(dst, dbf) = 0.312	MSU(dst, dmc, dbf) = 0.326	0.014
					MSU(dmc, dbf) = 0.043		
Pattern2	3	3	13	4, 6, 8, 13, 6, 3	MSU(dst, dbf) = 0.037	MSU(dst, dmc, dbf) = 0.301	0.176
				3, 5, 11, 6, 7, 18, 10	MSU(dmc, dbf) = 0.124		
	3	3	13	1, 2, 2, 2, 1, 2, 2, 1, 1, 1, 1, 1, 3	MSU(dst, dbf) = 0.152	MSU(dst, dmc, dbf) = 0.367	0.206
				(original observations)	MSU(dmc, dbf) = 0.161		
	3	3	13	Equal frequencies	MSU(dst, dbf) = 0.043	MSU(dst, dmc, dbf) = 0.326	0.186
					MSU(dmc, dbf) = 0.141		

*n* = Number of attributes; *c* = Cardinality of each attribute (all of them equal *c*); *k* = Number of record configurations in sample.

## Data Availability

For the simulation all data have been generated. Other data used is referenced in the text.
